# Impact of Drinking Water Quality on the Development of Enteroviral Diseases in Korea

**DOI:** 10.3390/ijerph15112551

**Published:** 2018-11-14

**Authors:** Yadav P. Joshi, Jong-Hun Kim, Ho Kim, Hae-Kwan Cheong

**Affiliations:** 1 Department of Social and Preventive Medicine, School of Medicine, Sungkyunkwan University, Suwon 440-746, Korea; yadavjoshi@gmail.com (Y.P.J.); kimjh32@skku.edu (J.-H.K.); 2 Department of Public Health, Manmohan Memorial Institute of Health Sciences, Kathmandu 44613, Nepal; 3 Asian College for Advance Studies, Lalitpur 44700, Nepal; 4 Department of Biostatistics and Epidemiology, Graduate School of Public Health, and Institute of Public Health and Environment, Seoul National University, Seoul 08826, Korea; hokim@snu.ac.kr

**Keywords:** enteroviruses, Poisson regression, temperature, water quality parameters, meta-analysis

## Abstract

Enterovirus diseases are fecal-orally transmitted, and its transmission may be closely related with the drinking water quality and other environmental factors. This study aimed to assess the association between environmental factors including drinking water quality and the incidence of enteroviral diseases in metropolitan provinces of Korea. Using monthly number of hand-foot-mouth disease (HFMD), aseptic meningitis (AM) and acute hemorrhage conjunctivitis (AHC) cases, generalized linear Poisson model was applied to estimate the effects of environmental factors on the monthly cases. An increase of mean temperature was associated with an increase of enteroviral diseases at 0–2 months lag, while an increase of turbidity was associated with increase in HFMD at 1 month lag and a decrease in AHC. An increase of residual chlorine in municipal drinking water was associated with a decrease in HFMD and AHC 2 and 3 months later. An increase of pH was associated with a maximum increase in AM 3 months later. The meta-analysis revealed the effects of the provincial and pooled variation in percent change of risks of environmental factors on HFMD, AM, and AHC cases at specific selected lags. This study suggests that the drinking water quality is one of the major determinants on enteroviral diseases.

## 1. Introduction

Enteroviruses are nonenveloped viruses which belong to the Picornaviridae family. They are grouped into polioviruses, coxsackievirus group A, coxsackievirus group B, echovirus, and the newer enterovirus serotypes 68 to 71 [1,2]. They are transmitted mainly by fecal–oral routes, or through direct contact with contaminated water, food, ophthalmic secretions, dermal lesions, or soil [1,3]. A variety of diseases are caused by different enteroviruses. Coxsackieviruses cause hand-foot-mouth disease (HFMD), aseptic meningitis (AM), acute hemorrhagic conjunctivitis (AHC), respiratory system infections, gastroenteritis, and heart diseases. Echoviruses are usually associated with AM, encephalitis, common cold, and respiratory diseases. The numbered enteroviruses (68 to 71) bring HFMD, bronchiolitis, conjunctivitis, meningitis and paralysis resembling poliomyelitis [1,2]. In East Asian countries including Korea, Japan, and Taiwan, HFMD, AM, and AHC are predominantly found during summer and early fall [4,5,6,7,8]. The seasonality of these enteroviral diseases suggests that meteorological variables and water quality parameters might influence the spread and distribution of enteroviruses [9,10].

In the Republic of Korea (Korea), enteroviral diseases are one of the major public health problems among children [11]. In a sentinel surveillance the Korea Centers for Disease Control and Prevention (KCDC) held from 2010 to 2013, 214,642 (0.53%) patients among 40,461,309 outpatient visits were clinically diagnosed with HFMD [8]. The outbreaks of AM in Korea occurred approximately every three years since 1990 [6,12]. Average annual incidence of AM among children under 15 was 3.5 per 1000 in 1996‒2001 [6]. During an outbreak in 2008, 67.7% of the samples from 758 enterovirus-positive patients were positive for AM, and of those, 98% were from children younger than 15 years [12]. The first known nationwide epidemic of AHC caused by coxsackievirus A24 occurred in Korea in 2002‒2003 with a September peak. Most of the patients were students under the age of 20 [5] and September was first month of a new semester after summer vacation.

Several environmental factors play an important role in the development, survival and transmission of enteroviruses. There was some suggestion on the potential role of drinking water in the transmission of enteroviruses [13,14,15]. The occurrence and survival of enteric viruses in water is influenced by temperature, suspended solids, turbulence, sunlight intensity, host excretion, nutrient content, and predation [16]. Weather variables also influence the transportation and dissemination of infectious agents, and might be important predictors of pediatric HFMD and AM occurrence in different regions [17,18,19]. Enteroviruses can survive for several days to months in room temperature in fomites, and aquatic environments [9,16,20]. They are inactivated by residual chlorine and sunlight in the water and inactivation is influenced by the pH and turbidity [21,22].

However, there are few epidemiological reports on the drinking water quality at national level. This study aimed at establishing the relationships between drinking water quality and monthly incidence of selected enteroviral diseases among the Korean metropolitan provinces while the seasonality of enteroviral diseases are considered.

## 2. Materials and Methods

### 2.1. Study Areas

Korea lies between the latitude of 33° N and 39° N, and the longitude of 124° E and 130° E. The elevation ranges up to 1950 m from the sea level with an average elevation of 282 m above the sea level. It is located in a transitional zone on the edge of the continental landmass of northeast Asia, bordering North Korea in the north and surrounded by water on three sides. Its total area is 99,617.38 km^2^ and 70% of which is occupied by mountains, while the southern and western parts of the peninsula have more plains. Administratively, it is comprised of seven metropolitan provinces and nine provinces. The total population is over 50 million with a population density of 511 persons per km^2^ with urbanization rate of 91.0% as of 2013 [23].

It shows complex climate characteristics, which represents both continental and oceanic climate. It has a temperate monsoon climate with cold winters, hot and humid summers, and sunny and generally dry springs and autumns. Summer arrives in late May with warm, moist prevailing winds from the Pacific Ocean. Relative humidity is the highest in July at 80–90 percent nationwide and the lowest in January and April at 30–50 percent. Rainfall during the summer is characterized by heavy showers with 60% of the annual rainfall is concentrated during three summer months from June to August. The rainy season typically starts from late June and lasts until late July.

### 2.2. Data Collection

Monthly reported cases of enteroviral diseases between January 2010 to December 2015 were extracted from the Healthcare Bigdata Hub, Health Insurance Review and Assessment Service (HIRA) [24]. Enteroviral diseases were defined based on the International Statistical Classification of Diseases and Related Health Problems 10th Revision’s (ICD-10) codes, B08.4 (HFMD), A87.0 (AM), and 30.3 (AHC). HIRA’s Healthcare Bigdata Hub is an application program interface that provides statistical information on diseases of public interest and contains information such as use of medical services, number of hospital visits, and use of medicines in the form of charts and graphs. Meteorological data, including the monthly average of daily maximum, minimum and mean temperatures (°C), relative humidity (%), rainfall (mm), sunshine (hours) and solar radiation (mJ/m^2^) were retrieved from the Korea Meteorological Administration [25]. Water quality data, including the monthly average of turbidity (NTU), and pH and residual chlorine (mg/L) of each water facility were obtained from the National Waterworks Information System (NWIS), Ministry of Environment [26]. NWIS manages the water quality data in real-time and discloses the information to the public. We averaged the water quality data among the water facilities in each province on monthly basis. Population data were retrieved from Statistics Korea [23].

### 2.3. Statistical Analysis

**Figure 1:**
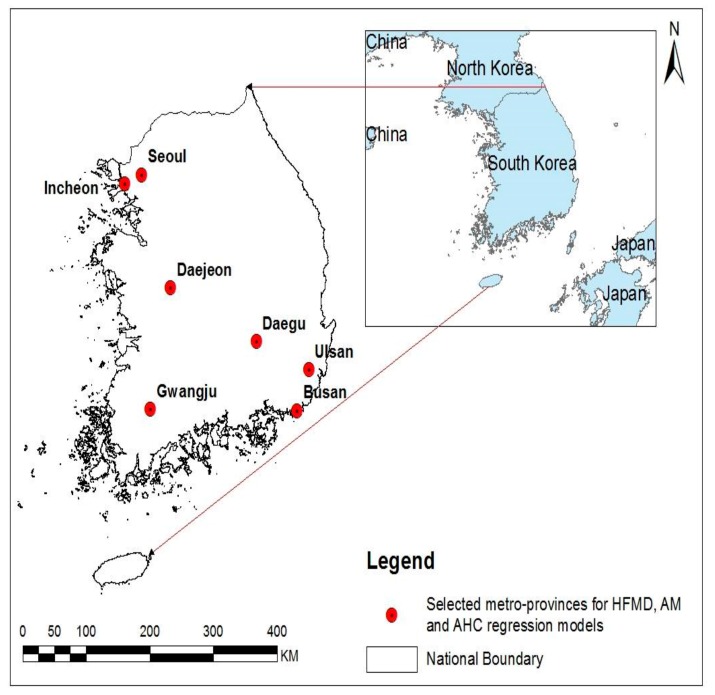
Map of geographical areas of Korea showing the six metropolitan provinces studied for model. The left panel shows the study sites in Korea map, and the right panel highlights the location of Korea in Asia.

Annual incidences per 100,000 were estimated. We performed a descriptive analysis on the distribution of weather, drinking water quality variables and incidence of enteroviral diseases in seven metropolitan provinces (Figure 1). In each metropolitan province, population was distributed in a relatively homogeneous pattern with an absolute majority of Korean ethnicity. Then, we plotted the time-series with the sum of enteroviral disease cases and average of environmental variables in seven provinces. We performed a univariate analysis between enteroviral disease cases and environmental variables using Spearman correlation coefficients. Among the meteorological variables, mean temperature was selected as an independent variable because it showed the highest correlation coefficients with selected enteroviral disease cases. In generalized linear model (GLM), multicollinearity was tested by using variance inflation factor (VIF) at an acceptable level (VIF ≤ 5) for selected explanatory variables [27]. Several studies have found the association between water quality factors and enteroviral diseases with different lags [7,8,18,19]. Therefore, after reviewing the survival of enteroviruses in an external environment and other possible effects of host and environment on the disease development, we designed the length of lag time in months [9,16,20], setting a maximum lag time of 4 months, lag 0 means number of disease cases and environmental factors in the given month while lag 4 indicates effect of environmental factors 4 months later on the development of enteroviral diseases.

#### 2.3.1. GLM Analysis

A GLM with a Poisson regression provided the best fit for the time series of over-dispersed data of disease cases and environmental factors [28]. Among the seven metropolitan provinces, a metropolitan province with the highest number of cases was initially selected and models were fitted for environmental factors [19]. Based on the correlation analysis and R^2^ values, regression models were developed using the variables significantly correlated with the diseases as explanatory variables and number of cases as the outcome variables. A forward stepwise approach was used in the regression model by adding the environmental parameters step by step until the best fitting model was found. Each model was adjusted separately to the year and meteropolitian provinces. The “log” annual province population as an offset, since the total population in metropolitan provinces had changed during the study period. Seasonality was controlled by including indicator variable for seasons. Linear models were diagnosed by the Akaike information criterion (AIC) to compare the model fit. The model with the smallest AIC fits the data better. The autocorrelation function of disease risk in each province was checked to find the significant lags for all provinces in GLM model. In the GLM analysis, the selected model was fitted to all metropolitan provinces in each month-lag. The model specifications we used for GLM are as follows.

Log (E(Y)) = β_0_ + β_1_ (mean temperature) + β_2_ (log (turbidity)) + β_3_ (log (residual chlorine/pH)) + offset (log (total population)) + factor (year) + factor (season) + factor (province) + ε_i_

where, E(Y) is the expected number of monthly case count; β_0_ is intercept; β_1_ and β_2_ are coefficients for mean temperature (in °C) and turbidity (in NTU) for HFMD, AM, and AHC; β_3_ is coefficient for residual chlorine (in mg/L) in HFMD and AHC while it is a coefficient for pH (in mg/L) in AM; factor (year) is an indicator variable for the years (2010 to 2015); factor (season) is an indicator variable (peak season = 1 other seasons = 0); factor (province) is an indicator variable for the metropolitian provinces of Korea and ε_i_ is error term. Each β represents increase of risk per one unit increase of each variable.

Percent change of risk and 95% CI were calculated by using a regression coefficient (β) and the following equation: percent change of risk = (exp[β] − 1) × 100 and 95% CI = (exp[β] − 1 ± 1.96 SE). Percent change of risk in mean temperature, turbidity, residual chlorine and pH indicates a change in enteroviral disease cases according to an increase of 1 unit. In turbidity, residual chlorine and pH, percent change of risk for 1 unit increase = [(1.01)^β^ − 1] × 100 and 95% CI = [(1.01)^β^ − 1 ± 1.96 SE].

#### 2.3.2. Meta-Analysis in the Seven Metropolitan Provinces

After estimating the effects of environmental variables on the development of enteroviral diseases in each metropolitan province by GLM, a meta-analysis was performed to obtain the overall risk that combined the results from different provinces. This result can be assumed as pooled effect of environmental factors on the development of enteroviral diseases in Korea. The specific lag with the highest effect of environmental variable was selected for each disease. It was assumed that the effect size of environmental variables on specific diseases in different lags could be different among the seven provinces, which were confirmed by testing heterogeneity (*I*^2^ derived from a chi-square test on Cochran’s Q statistic) of all the variables [29]. Thus, a restricted maximum likelihood (REML) estimation methods (one of the random effect models), rather than a fixed effect model was used to estimate the pooled effect [30]. In each model, a specific-lag heterogeneity analysis was performed for each environmental factor. The total heterogenicity of each environmental factor was found higher than 90 percent. Therefore, a sensitivity analysis was performed by comparing the total heterogeneity of each environmental factor in a selected model and total variability with the other models contained different populations with offset terms, seasons as indicator variables, or using natural spline function of the year at four degrees of freedom. Finally, the meta-analysis was conducted separately in each province. All statistical analysis was performed using R 3.3.3 (The R Foundation for Statistical Computing, Vienna, Austria) [31].

The protocol of this study was approved by the institutional review board of Sungkyunkwan University (2015-05-010), and informed consent was wavered according to the Privacy Protection Act of Korea.

## 3. Results

### 3.1. Descriptive Analysis

There were 1,722,863, 24,936 and 139,928 cases of HFMD, AM, and AHC cases, respectively, in Korea from January 2010 to December 2015 with annual average incidences of 573.4, 8.3 and 46.6 per 100,000 per year, respectively. The highest incidences of HFMD, AM, and AHC were reported in Ulsan (916.5), Gyeongnam (57.4) and Ulsan (166.9), respectively. Table 1 gives a general description of the enteroviral cases. Similarly, the higher incidences in 0–14 years per 100,000 were found in AM (110.6) and HFMD (12,189.5) than in age group 15 and above (AM = 48 and HFMD = 106). In contrast, higher incidences per 100,000 was reported in age group 15 and above (465.0) than incidence in age group 0–14 years (313.0) in AHC.

Descriptive statistics for HFMD, AM, and AHC cases and geographic and environmental variables in seven areas are shown in Table 2.

The monthly mean of daily mean temperatures was similar in all of the areas. Monthly averages of turbidity and residual chlorine were the highest in Incheon and the lowest in Seoul. The highest alkaline water was found in Seoul and the highest acidic water was found in Gwangju.

**Figure 2:**
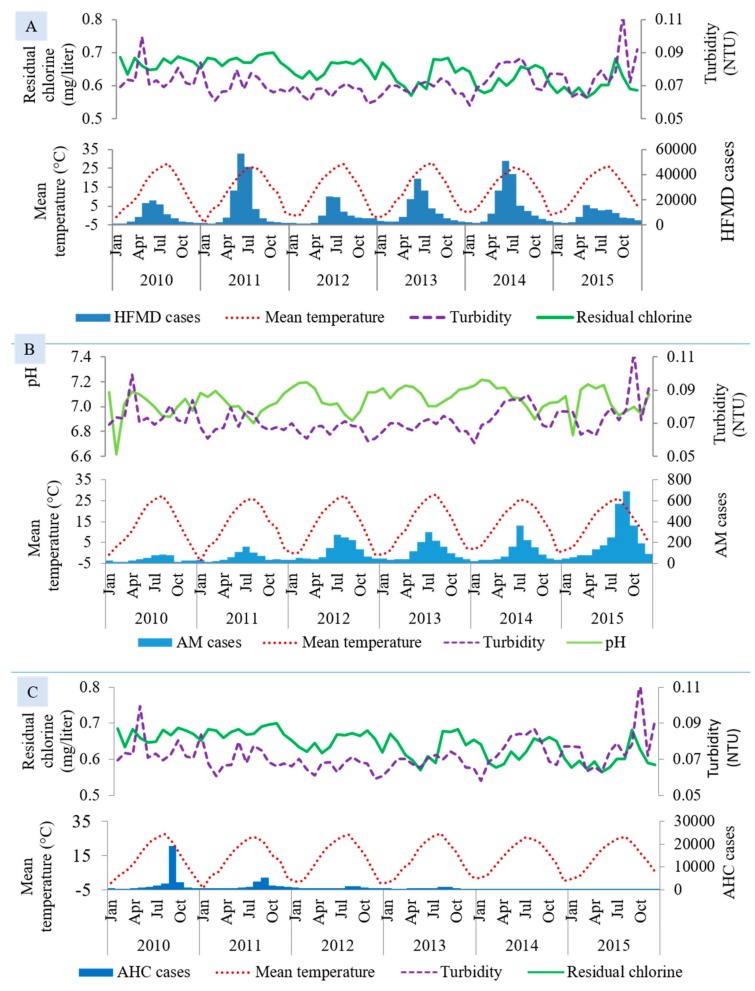
Monthly and yearly distributions of (**A**) HFMD, (**B**) AM, and (**C**) AHC cases with environmental factors in seven metropolitan provinces of Korea from 2010 to 2015. HFMD: hand-foot-mouth disease, AM: aseptic meningitis, AHC: acute hemorrhagic conjunctivitis.

Figure 2 summarizes the monthly variation of enteroviral disease cases with environmental variables in seven metropolitan provinces over the six-year period. Although HFMD, AM, and AHC cases occurred throughout the year, the number of cases rose during the summer months in HFMD and AM while the peaks in AHC were seen in September. The variations were found in the distribution of turbidity, residual chlorine and pH; however, mean temperature for AM and AHC peaked earlier than the peak of cases while in HFMD it peaked later. This figure also suggests the seasonal differences in the distribution of enteroviral diseases in Korea.

### 3.2. Generalized Linear Model

**Figure 3:**
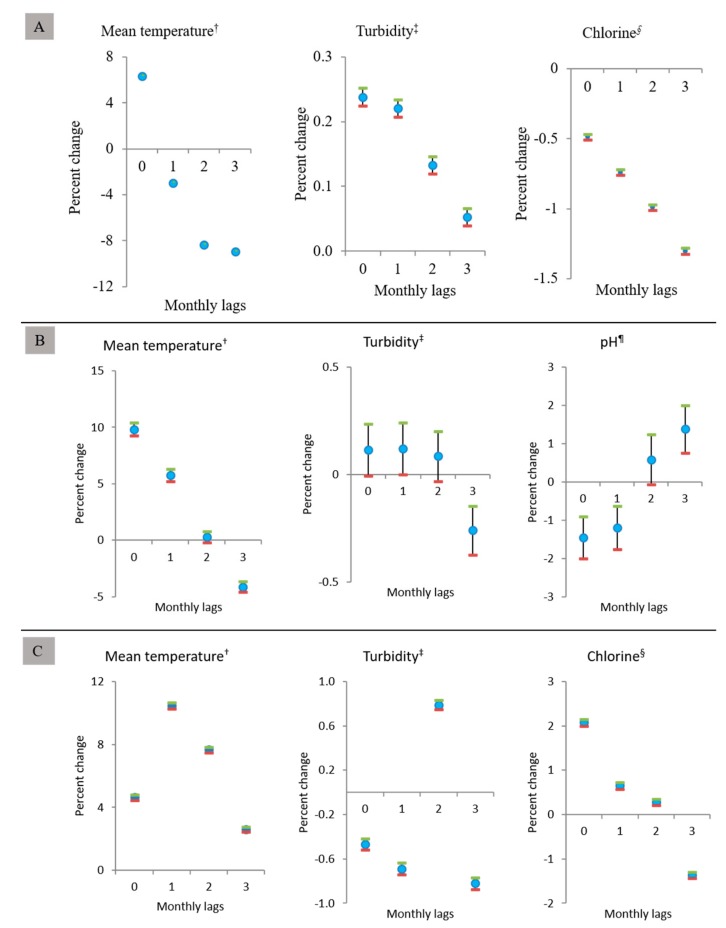
Poisson regression model showing associations between environmental variables and the monthly number of (**A**) HFMD, (**B**) AM, and (**C**) AHC cases from 2010 to 2015 in seven metropolitan provinces of Korea. ^†^ Monthly average of mean temperature (°C); ^‡^ monthly average of turbidity (NTU), ^§^ monthly average of residual chlorine (mg/L) and ^‡^ monthly average pH (mg/L). HFMD: hand-foot-mouth disease, AM: aseptic meningitis, AHC: acute hemorrhagic conjunctivitis.

HFMD, AM, and AHC disease cases for one month were correlated with the weather variables and water quality parameters during the same and preceding months. Generalized linear Poisson models were developed for each disease entity separately. The models demonstrate the associations between a single-month lag effect of the mean temperature, turbidity, residual chlorine/pH on the development of HFMD, AM, and AHC cases in all metropolitan provinces of Korea (Figure 3). The HFMD models (Figure 3A) suggest that a 1 °C increase in the mean temperature was associated with 6.29% (95% CI, 6.23–6.34%) increase in HFMD in the same months. An increase of 1 NTU in turbidity was also associated with 0.24% (95% CI, 0.22–0.25%) increase in HFMD in the same months and 1 mg/L increase of residual chlorine was associated with a decrease of −1.30% (95% CI, −1.32–−1.28%) in HFMD at a three-month lag. AM models (Figure 3B) suggest that an increase of 1°C in the mean temperature was associated with 9.83% (95% CI, 9.26–10.41%), increase in AM during the same months. An increase of 1 mg/L in pH was associated with a decrease of −1.45% (95% CI, −2.0–−0.91%) in AM at the same months. AHC models (Figure 3C) suggest that an increase of 1 °C in mean temperature was associated with an increase of 10.46% (95% CI, 10.27–10.65%), representing the maximum increase in AHC at one month’s lag. An increase of 1 NTU in turbidity was associated with a maximum decrease of −0.82% (95% CI, −0.87–−0.77%) AHC cases at three months later while an increase of 1 mg/L in residual chlorine was associated with an increase of 2.08% (95% CI, 2.0–2.16%) AHC cases in the same months.

### 3.3. Meta-Analysis

**Figure 4:**
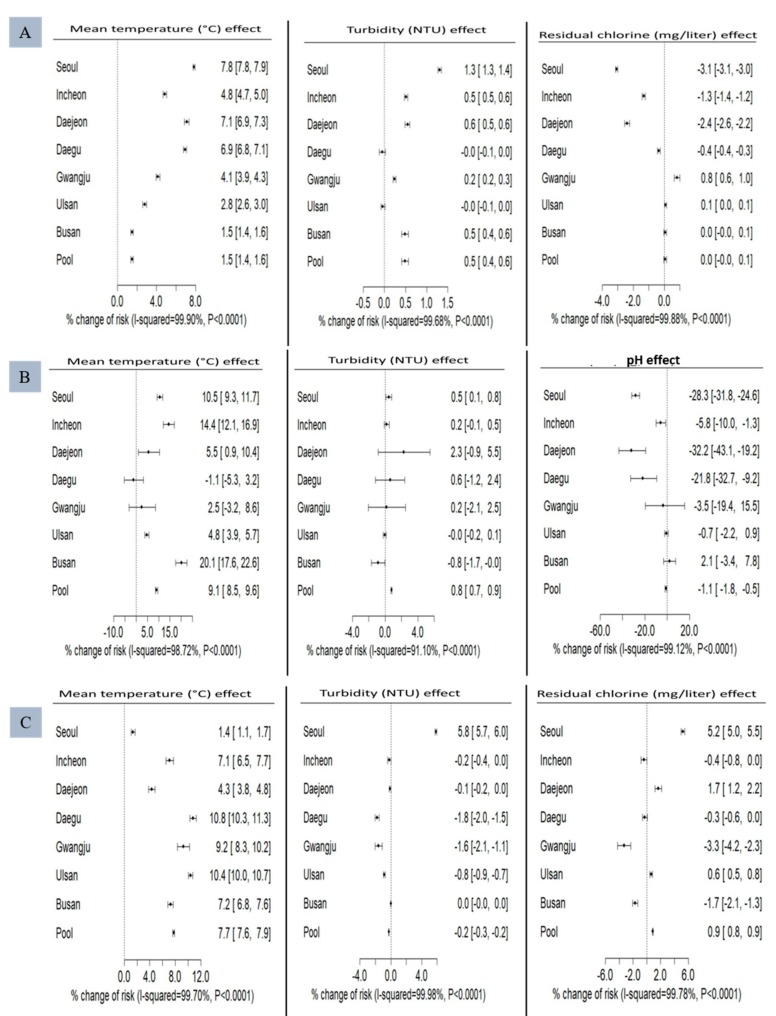
Percent change risk and meta-analysis for (**A**) HFMD, (**B**) AM, and (**C**) AHC cases from 2010 to 2015 in seven metropolitan provinces of Korea. HFMD: hand-foot-mouth disease, AM: aseptic meningitis, AHC: acute hemorrhagic conjunctivitis.

Figure 4 describes the percent changes in risk in the seven areas and the pooled effect via meta-analysis using an REML algorithm. The risk of HFMD for every 1 °C increase in mean temperature increased by 7.84% in Seoul at 0-month lag and the pooled effect of percent change risk was 1.49% (95% CI, 1.35–1.63%). The risk of HFMD rose to a maximum at lag 0 by 1.31% in Seoul per 1 NTU increase of turbidity and the pooled percent change in risk appeared to be 0.49% (95% CI, 0.41–0.57%). For residual chlorine, the maximum percent change with 1 mg/L increase at lag 3 was decreased by −3.07% in Seoul, and the pooled effect was 0.05% (95% CI, −0.05–0.14%) (Figure 4A). Similarly, the highest risk of AM at lag 0 increased by 20.07% for every 1 °C increase in mean temperature in Busan and the pooled effect percent change risk was 9.06% (95% CI, 8.50–9.63%). The pooled percent change in risk in every 1 NTU increase of turbidity at lag 3 was 0.80% (95% CI, 0.73–0.86%). As for pH, the decrease in percent change for an increase of 1 mg/L at lag 0 was −32.21% in Daejeon and the pooled effect was −1.14% (95% CI, −1.80–−0.84%) (Figure 4B). Likewise, the highest risk of AHC increased at lag 1 by 10.78% for every 1 °C increase in mean temperature in Daegu at lag 0 and the pooled effect percent change risk was 7.74% (95% CI, 7.62–7.86%). The maximum risk of AHC increased in Seoul at lag 2 by 5.85% per 1 NTU increase of turbidity and the pooled percent change in risk appeared to be −0.25% (95% CI, −0.27–−0.23%). As for residual chlorine, the maximum percent change for increase of 1 mg/L at lag 0 increased by 5.24%, in Seoul and the pooled effect was 0.88 (95% CI, 0.83–0.92%) (Figure 4C).

## 4. Discussions

During the six-year study period (2010–2015), the effects of air temperature and several water quality parameters on the development of three main enteroviral diseases, namely HFMD, AM, and AHC, in Korea were demonstrated. In Korea, the number of enteroviral disease cases and their associated incidences differ remarkably among the different provinces.

In metropolitan provinces, enteroviral diseases such as HFMD, AM, and AHC peaked in June, July and September, respectively. The enteroviral seasonal peaks were reported from various countries for different periods. For example, HFMD peaked in July in Japan [7], in May in Singapore [32], while a June peak was observed in Korea [8]. The AM peaks in July in Korea [6] and in May and June in Taiwan [4]. Likewise, the peaks for AHC in Korea were in September [5] while in India, it was during August and September [3]. Seasonal changes on enteroviral and other infectious diseases are characterized by the changes in the behaviors of their hosts. Seasonality in viral infection is a long-reorganized attribute that represents oscillation in pathogens and effective reproductive number, which in turn changes the infectivity, contact patterns, pathogen survival or host susceptibility but exact mechanism of seasonality is still unknown [33].

Most of the enteroviral incidences were in the 0–14 years old age group. Different age groups have different susceptibilities to infections; the clinical manifestations, severity of illness, and prognosis following the enteroviral infection also vary by age. This might be due to physiological and behavioral differences between children and adults. Infants and young children show a greater environmental exposure to enteric organisms than adults. They have yet to develop proper sanitary habits such as avoiding the consumption of dirty water during play. Neonates also get infections via vertical transmission [34,35]. Child care centers might also be fertile environments for the transmission of infectious disease pathogens, because young children lack appropriate hygienic behavior and they are naturally and immunologically more susceptible due to being grouped together in close proximity. In child care facilities, the risk of infectious diseases among children is dependent on the characteristics of a child including age, sex, immune status, and recent enrollment in a child care setting as well as characteristics of the care environment [34,35]. Thus, children who attend care centers are at increased risk for infectious diseases.

The prolonged survival of enteroviruses depends on environmental characteristics such as water sources, soil types, seasons, fomites, temperature, and relative humidity [9,18]. Therefore, the lagged effects of GLM reflected that each environmental variable’s delayed effects which are essential for the survival and proliferation of viruses in the external environment, seasonal fluctuations, human contact, outdoor activities and infectivity of pathogens to the human hosts. There was a positive association between the mean air temperature and enteroviral diseases. Previous studies from Korea, China, and Taiwan also reported a similar effect of temperature on enteroviral diseases [18,19,36]. Mean temperature had a decreased positive-lag relationship with enteroviral diseases. The survival of the virus in the external environment is decreased with the increase of time and the survival rate is inversely proportional to the level of temperature, and relative humidity [37]. Weather conditions could be associated with changes in human contact and behaviors. People are likely to spend more time out of the house in more crowded or air-conditioned environments during summer months, which could lead to increases in contact frequency among persons and accelerate enterovirus transmission.

Although enteroviruses are spread mainly through person-to-person contact, water-borne transmission may also occur [38], because an indirect transmission of viruses depends primarily on water. A case-control study in Korea found that consumption of unboiled drinking water and changes in water quality parameters were significantly associated with HFMD and AM transmission among children [15]. Similarly, another study conducted in 11 urban sites of Seoul metropolitan provinces in 1997‒1998 examined 23 tap water samples and among them 47.8% were positive for Coxsackievirus B and echovirus-6 [13]. An experimental U.S.-based study suggested that enteroviruses may be present in the water that meets acceptable limits of turbidity (<1.0 NTU), residual chlorine (>0.2 mg/L), and total coliform bacteria (<1 colony forming unit/100 mL) [39]. Because the water quality depends partly on land use and how water resources are managed and protected, these findings suggest the waterborne nature of enteroviral diseases.

In this study, turbidity showed positive effect on HFMD and both negative and positive relationship with AHC. Turbidity has a beneficial influence for virus survival. Drinking water turbidity in this study showed opposite associations with HFMD and AHC. This might be due to the different seasonal peaks of these two diseases as June is the peak month for HFMD and monsoon in Korea. During summer months, people may ingest more turbid water; additionally water intake related to recreational activities could be higher during that time than that of other seasons. The fluid consumption and water recreational activities would be lesser in fall months that in turn minimize the probability of HFDM and AM causing EVs. In contrast, the AHC peak is in September when water is less turbid and water-related recreational activities are less than those during summer months [20]. High turbidity alone may not account for the failure to remove viruses during water treatment [39]. The low level of chlorination in pools with high turbidity promotes the viral survival [21]. The sunlight inactivation of enteric viruses in natural environment is influenced by turbidity and presence of other chemicals [22]. Polioviruses, echoviruses, and coxsackievirus are inactivated to the same extent in presence of sunlight [22].

The GLM results of this study also show significant opposite association of HFMD and AHC cases with residual chlorine in Korea. Free water chlorine has the ability to alter the level of pollutants and kill harmful pathogens. Enteroviruses show a wide range of susceptibility to chlorine disinfection. The low level of chlorination and high turbidity can stimulate the survival of the virus [21]. An increase in the lag time for pH was associated to rise in the cases of AM. Enteroviruses show diverse adaptability against pH inside the host body versus being in the external environment. The rate of viral inactivation is greater at pH 6 than at pH 10 [40]. Salo et al. found the highest enteroviral inactivation rate at pH 3 and 9 which was enhanced due to the amount of sodium chloride present. Enteroviruses are more stable on acidic environment between 2 °C and 30 °C, because the temperature is so influential that the fastest inactivation rate at 2 °C is slower than the inactivation rate at 30 °C [41]. These findings suggest that enteroviral inactivation by residual chlorine and pH is influenced by the presence of several other factors and their quantities in an aquatic medium. The survival of enteroviruses in the external environment also depends on the local weather and the presence of temperature-dissolved oxygen in the water.

It is essential to conduct further detailed epidemiological and ecological studies on water quality parameters and their associated risks for enteroviral diseases in Korea. Enteroviral studies on socio-economic status and hygiene, mostly among the care centers in different regions, would also provide essential information on the disease transmission mechanism among the most vulnerable populations. Although there is little evidence that enteroviruses found in the environment are of public health importance, there are concerns about the possible dangers of contaminated sources, because enteroviruses are highly stable in water and, under certain conditions, are not completely eliminated by the treatment of drinking water [10].

The present study also has limitations. This is an ecological study and cannot explore individual-level associations. Our analysis was mainly exploratory. Furthermore, we used data from only the metropolitan provinces of Korea, which are not representative of the whole country. We used the air temperature instead of water temperature. Water supply to the study areas is mostly from the large-scale municipal supply, the quality of which is quite well controlled. Therefore, water may not represent the main mode of exposure in these populations.

In this study, the different direction of the effect size of a specific environmental factor may reflect the different distribution of the basic mechanism of the selected enteroviral disease development in the local area. There were variable lag-effects of weather factors and water quality parameters in the development of enteroviral diseases in study area. Thus, through the pattern of response to the specific environmental factors on the enteroviral disease risk, we may accept or reject the specific determinants of enteroviral disease development in the area. The heterogeneity of direction and the size of response to the specific environmental factors in a specific province indicate the different distribution of the main determinants of the risk of enteroviral disease development in the area. The meta-analysis in this study can provide a potent insight on the impact of environmental factors for on the enteroviral diseases effect when local and national variability are considered. Comparison across the seven metropolitan provinces is also important for demonstrating effects from one province to another.

## 5. Conclusions

The associations between environmental factors and enteroviral disease case can be widely variable according to locations, local climate factors, and water quality parameters. This study contributed to the current research by improving the understanding of relationship between the variations of environmental factors and enteroviral diseases. The findings of meteorological factors and enteroviral diseases show the impact of climate variation on the development of enteroviral diseases in metropolitan provinces of Korea.

## Figures and Tables

**Table 1:** General description of enteroviral disease cases from 2010 to 2015 in Korea.

Variables		No. of Cases			Incidence (/100,000/year)		
		HFMD	AM	AHC	HFMD	AM	AHC
Year	2010	185,560	3050	56,878	375.6	6.2	115.1
	2011	380,111	3499	42,710	763.6	7.0	85.8
	2012	226,650	4344	18,491	453.3	8.7	37.0
	2013	305,536	4287	12,692	608.4	8.5	25.3
	2014	411,191	3493	4976	815.5	6.9	9.9
	2015	213,815	6263	4181	422.4	12.4	8.3
Metropolitan provinces *	Seoul	278,144	2601	37,572	465.5	4.4	62.9
	Busan	107,852	1117	12,609	523.6	5.4	61.2
	Daegu	100,515	130	6146	679.4	0.9	41.5
	Incheon	74,639	595	2981	443.9	3.5	17.7
	Gwangju	68,518	77	1365	757.3	0.9	15.1
	Daejeon	50,437	210	5620	548.9	2.3	61.2
	Ulsan	61,632	3195	11,223	916.5	47.5	166.9
	Gyeonggi	453,361	4511	25,022	628.3	6.3	34.7
	Gangwon	51,675	105	1569	575.1	1.2	17.5
Nonmetropolitan provinces	Chungbuk	56,101	210	1823	604.8	2.3	19.7
	Chungnam	73,130	689	3746	562.2	5.3	28.8
	Jeonbuk	66,476	150	6742	616.2	1.4	62.5
	Jeonnam	52,582	100	1930	496.5	0.9	18.2
	Gyeongbuk	81,073	30	5285	512.3	0.2	33.4
	Gyeongnam	129,494	11,201	14,937	664.1	57.4	76.6
	Jeju	17,234	15	1358	507.3	0.4	40.0
Total		1,722,863	24,936	139,928	573.4	8.3	46.6

HFMD: hand-foot-mouth disease, AM: aseptic meningitis, AHC: acute hemorrhagic conjunctivitis, Metropolitan provinces *: One special city and six metropolitan cities.

**Table 2:** Descriptive statistics of the seven metropolitan provinces in Korea, 2010–2015 (mean ± standard deviation).

Variables	Seoul	Incheon	Daegu	Gwangju	Daejeon	Busan	Ulsan
HFMD	3863.1 ± 5165.9	1036.7 ± 1366.4	1396.0 ± 1810.6	951.6 ± 920.1	700.5 ± 855.0	1497.9 ± 1809	856 ± 883.4
AM	36.1 ± 50.6	10.8 ± 21.7	2.1 ± 2.0	2.1 ± 1.5	6.2 ± 9.2	19.3 ± 26.8	44.4 ± 33.9
AHC	521.8 ± 1619.0	41.4 ± 46.1	85.4 ± 169.2	19.2 ± 37.5	78.1 ± 110.0	175.1 ± 334.4	155.9 ± 213.8
Population density ^†^	16,433.8 ± 120.0	2904.1 ± 63.6	2783.0 ± 7.5	3010.0 ± 19.1	2836.3 ± 21.6	4516.7 ± 30.9	1061.3 ± 16.7
Mean temperature ^‡^	13.52 ± 9.25	13 ± 8.95	14.64 ± 9.28	14.21 ± 9.17	13.55 ± 9.31	14.99 ± 8.05	14.38 ± 8.53
Turbidity ^¶^	0.04 ± 0.04	0.10 ± 0.03	0.07 ± 0.01	0.06 ± 0.04	0.06 ± 0.02	0.09 ± 0.02	0.08 ± 0.02
Residual chlorine ^§^	0.49 ± 0.07	0.78 ± 0.07	0.55 ± 0.06	0.62 ± 0.04	0.72 ± 0.06	0.58 ± 0.06	0.76 ± 0.15
pH ^§^	7.13 ± 0.09	7.36 ± 0.47	7.10 ± 0.17	6.77 ± 0.12	7.08 ± 0.10	6.84 ± 0.14	7.12 ± 0.19

HFMD: hand-foot-mouth disease, AM: aseptic meningitis, AHC: acute hemorrhagic conjunctivitis, Units: ^†^ persons/Km^2^; ^‡^ °C/month; ^¶^ NTU, ^§^ moles/L.

## Abbreviations

AHC	acute hemorrhagic conjunctivitis
AIC	Akaike information criterion
AM	aseptic meningitis
GLM	generalized linear model
HFMD	hand-foot-mouth disease
HIRA	Health Insurance Review and Assessment Service, Korea
NTU	nephelometric turbidity unit
NWIS	National Waterworks Information Service, Korea
REML	restricted maximum likelihood
VIF	variance inflation factor
